# Testing the reproductive and somatic trade‐off in female Columbian ground squirrels

**DOI:** 10.1002/ece3.2215

**Published:** 2016-10-04

**Authors:** Kristin Rubach, Mingyan Wu, Asheber Abebe, F. Stephen Dobson, Jan O. Murie, Vincent A. Viblanc

**Affiliations:** ^1^ Department of Biological Sciences Auburn University Auburn Alabama 36840; ^2^ Department of Mathematics and Statistics Auburn University Auburn Alabama 36840; ^3^ Department of Biological Sciences University of Alberta Edmonton Alberta T6G 2E9; ^4^ Département Ecologie, Physiologie et Ethologie (DEPE) Institut Pluridisciplinaire Hubert Curien (IPHC) Université de Strasbourg 23 rue Becquerel 67087 Strasbourg France; ^5^ Centre National de la Recherche Scientifique Unité Mixte de Recherche 7178 67087 Strasbourg France

**Keywords:** Capital breeding, Columbian ground squirrels, energy allocation, income breeding, reproductive allocation

## Abstract

Energetic trade‐offs in resource allocation form the basis of life‐history theory, which predicts that reproductive allocation in a given season should negatively affect future reproduction or individual survival. We examined how allocation of resources differed between successful and unsuccessful breeding female Columbian ground squirrels to discern any effects of resource allocation on reproductive and somatic efforts. We compared the survival rates, subsequent reprodction, and mass gain of successful breeders (females that successfully weaned young) and unsuccessful breeders (females that failed to give birth or wean young) and investigated “carryover” effects to the next year. Starting capital was an important factor influencing whether successful reproduction was initiated or not, as females with the lowest spring emergence masses did not give birth to a litter in that year. Females that were successful and unsuccessful at breeding in one year, however, were equally likely to be successful breeders in the next year and at very similar litter sizes. Although successful and unsuccessful breeding females showed no difference in over winter survival, females that failed to wean a litter gained additional mass during the season when they failed. The next year, those females had increased energy “capital” in the spring, leading to larger litter sizes. Columbian ground squirrels appear to act as income breeders that also rely on stored capital to increase their propensity for future reproduction. Failed breeders in one year “prepare” for future reproduction by accumulating additional mass, which is “carried over” to the subsequent reproductive season.

## Introduction

In order to survive and reproduce, animals must acquire energy from the environment and successfully allocate it to various metabolic needs (Brown et al. 2004). However, because energy resources are usually limited under natural conditions, life‐history traits are seldom maximally expressed (Fisher 1930; Reznick 1985; Kunz and Orrell 2004). Resources allocated to one biological function often reduce availability for allocation to other biological functions, a type of life‐history trade‐off (Stearns 1992).

An assumption of life‐history theory is the existence of “energy costs” associated with trade‐offs with respect to survival and reproduction (Lack 1966; Williams 1966; Hirshfield and Tinkle 1975; Bell 1980; Stearns 1992). In iteroparous species (i.e., those that reproduce more than once), annual resource allocation can be divided into two primary biological functions: somatic and reproductive efforts (Hirshfield and Tinkle 1975). Resources can be allocated either to individual soma in the form of growth, personal maintenance, and survival, or to reproduction in the form of immediate offspring production and parental care. When environmental resources are acquired annually or when the annual energy budget is fixed, these two categories add up to the total energy resources available for allocation. If current reproduction requires a high level of allocation, females must reallocate energy from growth and somatic maintenance to accommodate this need, giving rise to the idea that reproduction might entail costs, as somatic development (maintenance) and/or future reproduction may be reduced (Williams 1966; Partridge and Harvey 1985; Reznick 1985; Partridge 1992).

One way to study somatic and reproductive allocation is to focus on the ways in which resources are accumulated (Jönsson 1997; Houston et al. 2007). In instances where stored resources are devoted to reproduction, a species is considered a “capital breeder” under the analogy that saved capital is “funding” reproduction. In contrast, when reproduction relies on the daily accumulation of resources, the species is considered an “income breeder” (Jönsson 1997). In the latter case, females faced with increased reproductive demands may either reallocate somatic resources to reproduction or augment their energy income by increased foraging (Van Noordwijk and DeJong 1986). In ground squirrels, females in good body condition (i.e., high body mass) allocate more resources to reproduction in the form of larger or heavier litters than females in poor condition (Murie and Dobson 1987; Michener 1989; Dobson et al. 1999; Risch et al. 2007). Females in good condition may also show improved survival and concurrent subsequent breeding through increased somatic effort. Changes in resource allocation might involve costs in terms of future survival or reproduction by the parent (Williams 1966; Fisher and Bloomberg 2011), or costs may be defrayed onto offspring if the increasing demands on the parent are not met elsewhere (Morris 1986).

Columbian ground squirrels (*Urocitellus columbianus*) are predominantly income breeders with a short active season of 3–4 months in which major breeding events (i.e., mating, gestation, and lactation) occur within a brief time period coinciding with seasonal environmental changes (Murie and Harris 1982; Dobson et al. 1992; Broussard et al. 2005). Virtually, all females attempt breeding during their prime reproductive years (ages 3–7); while some are successful at weaning litters, others are not (Dobson and Kjelgaard 1985; Dobson and Murie 1987; Dobson 1988; Broussard et al. 2003, 2008). Although stored capital in the form of body mass has a significant influence on ground squirrel reproduction, energy allocated to young during gestation and lactation comes primarily from daily resource acquisition (Risch et al. 1995; Dobson et al. 1999; Broussard et al. 2005). Therefore, the balance of resources between somatic and reproductive allocations might be especially important for adult females in this species.

Comparisons between females that successfully breed and females that are not successful at breeding can be used to test for energy allocation shifts between reproduction and somatic maintenance. Murie and Dobson (1987) and Neuhaus (2000) found that survival did not vary for mothers with different levels of reproductive effort, as reflected by litter size. These authors suggested that an association of body condition and reproduction might explain the apparent lack of phenotypic costs of reproduction, so that costs were masked by differences in resource accumulations among mothers. A similar mechanism might explain the lack of phenotypic costs in experimental studies of litter manipulation. For instance, neither experimental increases nor reductions in litter size affected maternal survival or future reproduction (Hare and Murie 1992; Skibiel et al. 2013). Both types of manipulation, however, affected mothers’ body mass at subsequent spring emergence such that females with lower levels of reproductive allocation were heavier.

If reproductive costs exist, they are likely to be most evident in a comparison of individuals of different reproductive status. Females that do not produce a litter have little or no current reproductive effort and should thus provide an excellent model for examining the ramifications of reproduction. If they carry stored resources over into the following year, any additional body mass might have a positive influence on reproductive success the subsequent year. Spring body mass at emergence from hibernation has a significant positive influence on the production of offspring, both in terms of numbers and quality (Risch et al. 1995; Dobson et al. 1999; Skibiel et al. 2009). Thus, improved body condition associated with failed breeding is expected to increase a female's capacity for future reproduction.

The purpose of our study was to examine how allocations of resources differed between naturally successful and unsuccessful breeding female Columbian ground squirrels during a 24‐year period, thus allowing us to discern the effects of resource allocation to reproductive and somatic efforts under natural conditions as opposed to artificial manipulations of resource allocation. We hypothesized that reproduction should have negative influences on the future survival and reproduction of adult females, as a result of higher resource allocation to offspring rather than personal soma in a given year. Thus, we expected successfully reproducing females (i.e. females that successfully weaned a litter) to have lower future survival and reproduction than females that did not reproduce. Alternatively, if breeding females were able to compensate by increasing their daily intake of resources, we would expect them to show no “cost of reproduction” in terms of survival or reproduction in the following year.

Two hypotheses might explain the energy fate of unsuccessful breeding. First, females that did not successfully breed may have allocated more resources to somatic development. We would then expect unsuccessful females to show a greater increase in body mass and higher survival rates to the subsequent year compared to co‐occurring breeding females (e.g., Clutton‐Brock 1984; Festa‐Bianchet et al. 1998; Fisher and Bloomberg 2011; Rughetti et al. 2015). Alternatively, if little or no difference in body mass and survival to the following year were found between successful and unsuccessful breeders, this would suggest that unsuccessful females are not shifting resources to somatic effort (e.g., Millar et al. 1992), but rather decreasing their overall resource income. Thus, there might be no benefit to somatic allocation that would offset the lack of reproductive allocation in the current year. In this case, failure to reproduce would not enhance future reproductive value, and females may be using a “best of a bad job” strategy, “waiting” until the next year to attempt reproduction once again.

## Materials and Methods

### General methods

Columbian ground squirrels are semifossioral hibernating rodents that live in subalpine and alpine meadows in the Rocky Mountains of the northwestern USA and southwestern Canada. We studied them from 1992 to 2015 in the Sheep River Provincial Park, Alberta, Canada (50 39° 7′ N, 114 37° 27′ W; 1550 m elevation). These ground squirrels emerge from hibernation in late April to early May and have an active season that extends into early August. We trapped ground squirrels as they emerged from hibernation in the spring, using live traps (13 × 13 × 40 cm; Tomahawk, WI) baited with peanut butter. At the time of trapping, each squirrel was weighed to the nearest 5 g using a Pesola spring scale (Pesola Ag, Baar, Switzerland). The ground squirrel was then given a unique ear tag number (#1‐Monel metal; National Band and Tag Company, Newport, KY) and a unique mark for visual identification with black hair dye (Clairol, Stamford, CT). The young first emerged from nest burrows at the time of weaning in mid June to early July. At that time, adult females were caught with their litters and the young were ear tagged and given unique dye markings.

Behavioral observations were taken daily from 3‐m tall wooden stands. Mating dates for females were determined from the occurrence of above‐ and belowground consortships with males (Raveh et al. 2010, 2011). From these dates, parturition and weaning dates for litters could be estimated. When mating date of a female was undetected, the condition of the vulva and presence of copulatory plug material in the vulva, sperm, and copulatory plug material on the fur were used as indicators of successful mating (Murie and Harris 1982). Following methods developed by Hare and Murie (1992), we trapped females 2–3 days before their expected parturition date, about 22 days after mating (Shaw 1925; Murie and Harris 1982; Murie 1992), brought the females into an on‐site laboratory, and housed them in polycarbonate microvent rat cages (267 × 483 × 200 mm; Allentown Caging Equipment Company, Allentown, NJ). They were given wood shavings and newspaper as nesting material, and apple, lettuce, and horse feed (EQuisine sweet show horse ration; Unifeed, Okotoks, Alberta, Canada) were provided ad libitium. At parturition, mothers (nearest 5 g) and pups (nearest 0.01 g) were weighed. Pups were sexed and marked with a small tissue biopsy by clipping a toenail bud as previously described by Hare and Murie (1992). Mothers and neonatal young were released approximately a day after birth into nest burrows. These nest burrows were previously known from observations of females entering them with loads of nest material (natural dry grass from the meadow). If a female did not give birth in the laboratory after approximately 7–10 days, she was examined for the presence of mammary tissue and released at her original capture location.

In the 24‐year data set, 1992–2015, we recorded the life histories of females that lived to be at least 2 years old, the most likely age at which they become reproductively mature (Dobson and Murie 1987). Few females breed as 1‐year‐olds (*N* = 11), as yearling females are still growing and are of relatively low body mass (Dobson and Murie 1987; Dobson 1992). Two‐year‐old females that failed to reach reproductive maturity were also still growing (Broussard et al. 2008) and thus may have exhibited different patterns of somatic allocation from fully grown adults. We thus restricted our analyses to females that were ≥3 years old (*N* = 137), all of whom mated and had the opportunity to reproduce. When older than 9 years of age, females (*N* = 6) exhibited evidence of senescence (losses in maternal body mass or extremely low litter sizes) and we excluded these cases from the analyses.

Females can breed successfully in some years but not in others; thus, the reported sample sizes are cumulative for each breeding status. Females that produced a litter and weaned pups within a particular year were considered reproductively successful and classified as breeders (*N* = 321). Reproductively unsuccessful females (“failed to wean offspring,” *N* = 99) were females that mated but either did not give birth in the laboratory (“failed during gestation,” *N* = 34) or gave birth but were unsuccessful at weaning young (“failed during lactation,” *N* = 41). Because we did not know when some mothers failed at reproduction (*N* = 24), we pooled failed breeders for some analyses. Lactation is a highly demanding period in terms of energy expenditure (Clutton‐Brock et al. 1989; Robbins 1993; Speakman 2008; in ground squirrels, Skibiel et al. 2013). Thus, the comparison of successful breeders (viz., those that weaned young), females that failed to give birth, and females that failed to wean offspring allowed us to examine how resources were allocated between successful females and those that did not allocate resources to offspring during at least part of the lactation period.

### Somatic and reproductive allocations

Somatic allocation was estimated by measuring female body mass at two different times during the active season, at emergence from hibernation and at weaning. When young first emerge from natal nest burrows, lactation is essentially completed (Murie and Harris 1982) and the resource commitment to offspring has virtually ceased (Mattingly and McClure 1982; Kenagy et al. 1989; Michener 1989). Body mass of unsuccessful breeding females was also measured at the time that they would have weaned a litter had they been successful (i.e., about 52 days after mating, Murie and Dobson 1987). Mass at emergence the subsequent year was used to determine whether females that failed started the next year with more capital and whether they had a greater likelihood to successfully reproduce in the subsequent year than previously successful females (Broussard et al. 2005). Because samples were limited, we also examined female body mass dynamics (mass gain or loss) between spring emergence in a given year to spring emergence in the next (viz., “carryover” effects in body mass). Reproductive allocation was estimated from the presence or absence of a litter in the present year, and “carryover” effects were investigated by considering the size at birth of the subsequent litter, the year *after* a female was successful or not.

### Statistics

We tested for the effects of female breeding status and mass at weaning (independent variables) on her survival (dependent variable). Multivariate Cox regression models were used to analyze survival between when females entered our study and death (binomial factor, 0 = present in study, 1 = death). Breeding status (successful and unsuccessful) and weaning mass were included as predictor variables, with age as a time‐varying covariate and year as a random variable (see below) (Cox and Oakes 1984). A maximum‐likelihood fit of the model was obtained via simultaneous maximization of the integrated partial likelihood (Ripatti and Palmgren 2000) over the fixed effects and the random effect covariance parameters. We further examined the effect of female breeding status on female mass using linear mixed models. We thus regressed female mass at weaning (or theoretical weaning mass for failed females) on breeding status (i.e., successful, failed during gestation or failed during lactation). Year and female identity were included as random variables to account for among‐year variability and intrinsic differences in female quality (females repeated in several years). Female Columbian ground squirrels are highly philopatric, and emigration by adults is rare (Wiggett and Boag 1992; Neuhaus 2006; Arnaud et al. 2012), and thus, any disappearance from the population was most likely due to death.

When analyzing somatic and reproduction allocation, linear mixed models were implemented in R (version 3.2.2) using the lme4 package (Bates et al. 2015; R Core Team 2013). Again, we included individual identification number and year as random variables in our models. Emergence mass of adult females (in the current and subsequent year), weaning mass, and litter size at birth were normally distributed as evident by visual inspections of histograms of residuals, so we used Gaussian distributions for modeling these variables. Coefficients of determination (conditional *R* ^2^ values) were calculated following Nakagawa and Schielzeth (2013).

The influence of breeding status in one year on the probability of breeding successfully in the next year was examined using Markov transition mixed models (Diggle et al. 2002) in which breeding status in the second year was regressed on breeding status in the first year, with spring body mass in the second year as a covariate and year as a random variable, and applying a binomial error term. Future litter size (in the next year) was compared among females of different breeding status using linear models and Tukey post hoc tests. Finally, the relationship between litter size and body mass was examined in mixed models where female identity and year were random variables, and conditional coefficients of determination were used to estimate effect sizes.

## Results

### Survival

Females that failed during gestation suffered poorer survival to the next spring than females that failed to wean a litter and those that weaned litters (by 11.2% and 12.2%, respectively; Table 1). These differences were not significant, likely due to more limited samples of females that failed during gestation and lactation (*N* = 34 and 41, respectively, *N* = 321 for breeders; mixed model with year and female identity as random variables, $R_{\mathrm{conditional}}^{2}$ = 0.232, likelihood ratio test, χ^2^ = 2.41, *P *=* *0.49). Reproductive status (successful vs. unsuccessful) and mass at weaning in a given year did not significantly influence female survival to the next year (Cox regression with binomial error, random year, *R* ^2^ = 0.324; breeders *N* = 321, failed breeders *N* = 75; likelihood ratio test, χ^2^ = 1.71, *P *=* *0.19). Including mass gained from 1st year emergence to either subsequent weaning time or 2nd year emergence had trivial effects on the model.

**Table 1:** Survival of reproductively mature (≥3 years old) female Columbian ground squirrels belonging to breeder or nonbreeder classes

Breeding status	Average survival (%)	*N*	*P*
Breeders	76.9 ± 2.4	321	0.39^a^
Failed at birth	64.7 ± 8.3	34	0.70^b^
Failed at weaning	75.7 ± 6.8	41	0.99^c^

Average survival listed with standard errors. Significance based on Tukey post hoc test.

a Comparison of breeders and failed at birth.

b Comparison of failed at birth and failed at weaning.

c Comparison of breeders and failed at weaning.

### Body mass and carryover effects

**Figure 1:**
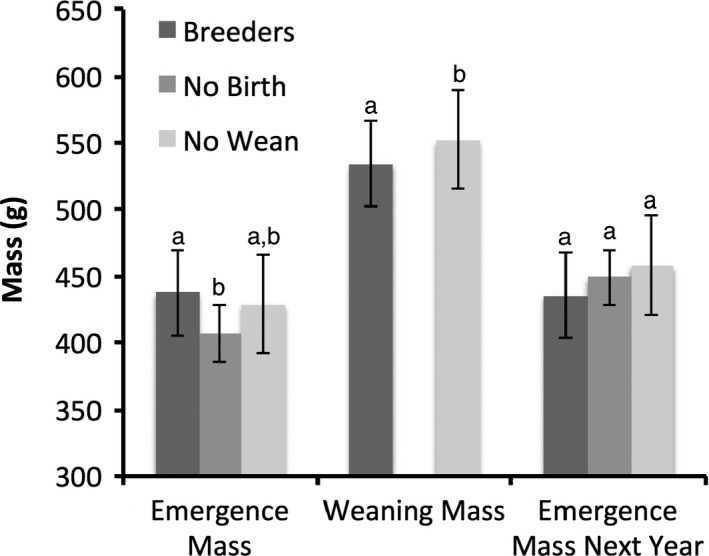
(1) Spring emergence mass of female Columbian ground squirrels (3 years of age and older) in the current year (successful breeders, *N* = 319, failed at birth, *N* = 34, failed to wean, *N* = 39); (2) weaning mass of females in the current year (successful breeders, *N* = 313, failed to wean, *N* = 15); and (3) spring emergence mass the next year (successful breeders in the previous year, *N* = 218, previously failed at birth, *N* = 20, previously failed to wean, *N* = 27). Significance determined with Tukey's HSD test and indicated by different letters. Results are given as means ± SD

At emergence from hibernation in the spring, body mass varied with the reproductive success of adult females (Fig. 1; mixed model, year and female identity as random variables, breeding status as a fixed factor; $R_{\mathrm{conditional}}^{2}$ = 0.646; likelihood ratio test, χ^2^ = 14.7, *P *=* *0.002, *N* = 392). Females that subsequently failed during gestation were 7.0% lighter than females that successfully weaned offspring (406.9 ± 6.9 g, *N* = 34, and 437.5 ± 2.7 g, *N* = 319, respectively; Tukey difference = −30.60 g, 95% CI = −52.26 to −8.94 g, *P *=* *0.002). At the same time, females that subsequently failed during lactation were only 2.0% lighter in body mass to those that later successfully weaned offspring (429.0 ± 6.7 g, *N* = 39, and 437.5 ± 2.7 g, *N* = 319, respectively; Tukey difference: = −8.53 g, 95% CI = −28.90 to 11.83 g, *P *=* *0.70).

For analyses of body mass at weaning, due to low sample size, we pooled females failing during gestation and lactation. Unsuccessful females were 3.3% heavier than successfully breeding females (Fig. 1; mixed model, year and female identity as random variables, breeding status as a fixed factor; $R_{\mathrm{conditional}}^{2}$ = 0.565; likelihood ratio test, χ^2^ = 13.7, *P *=* *0.0002, *N* = 328; mean mass = 551.9 ±12.4 g, *N* = 15, and 534.4 ± 2.9 g, *N* = 313, respectively). The former gained 23.5% more body mass over the reproductive season than mothers that successfully weaned offspring (mixed model, year and female identity as random variables, breeding status and spring emergence mass as fixed factors; $R_{\mathrm{conditional}}^{2}$ = 0.511; likelihood ratio test for breeding status, χ^2^ = 16.8, *P* < 0.0001, *N* = 327; mean mass gain = 120.9 ± 13.5 g, *N* = 14, and 97.9 ± 2.9 g, *N* = 313, respectively).

At emergence from hibernation in the following spring, successful breeders were 3.7% lighter than females that failed during gestation (Fig. 1, 435.6 ± 2.9 g, *N* = 218, 452.3 ± 12.7 g, *N* = 20, respectively, Tukey difference = 16.63, 95% CI = −11.2 to 44.4 g, *P *=* *0.41). Females that failed during lactation were 1.3% heavier than those who failed during gestation (458.0 ± 11.0 g, *N* = 27, 452.3 ± 12.7 g, *N* = 20, respectively, Tukey difference = 5.7, 95% CI = −29.4 to 40.8 g, *P *=* *0.97). Emergence mass in the next year for females that failed during lactation were 5.0% heavier and approached significance compared to successful breeders (458.0 ± 11.0 g, *N* = 27, 435.6 ± 2.9 g, *N* = 218, respectively, Tukey difference = 22.3, 95% CI = −1.9 to 46.6 g, *P *=* *0.08). Due to low sample sizes and to examine this difference further, we pooled females that had failed during gestation or lactation. Those that were successful at weaning litters were significantly lighter in body mass compared to those that failed either at gestation or lactation (mixed model, year and female identity as random variables, breeding status as a fixed factor; $R_{\mathrm{conditional}}^{2}$ = 0.728; likelihood ratio test, χ^2^ = 37.6, *P *<* *0.0001, *N* = 265).

Between the time of offspring weaning in a given season and the following spring, females that failed to wean lost significantly more body mass than females successfully raising a litter (mixed model, year and female identity as random variables, breeding status and body mass at the time of weaning as fixed factors; $R_{\mathrm{conditional}}^{2}$ = 0.598; likelihood ratio test for breeding status, χ^2^ = 4.2, *P *=* *0.04, *N* = 222; mean mass loss = 108.9 ± 19.0 g, *N* = 9, and mean mass loss = 97.9 ± 3.6 g, *N* = 213, respectively).

Females failing to wean offspring in a given year exhibited 11.1% increase in body mass the following spring, whereas successful breeders lost 0.4% (mixed model, year and female identity as random variables, breeding status and previous spring emergence mass as fixed factors; $R_{\mathrm{conditional}}^{2}$ = 0.520; likelihood ratio test for breeding status, χ^2^ = 16.8, *P *<* *0.0001, *N* = 243; mean mass gain = 28.5 ± 6.7 g, *N* = 26, and mean mass gain = −2.0 ± 2.7 g, *N* = 217, respectively).

### Reproductive success

Successful breeding in a given year did not depend on the previous breeding outcome (Table 2). The proportion of females from each reproductive status that successfully bred in the following year did not differ significantly (Fisher exact test, *N* = 283, *P *=* *0.49). Litter size at birth in the following year was not significantly different for reproductively successful and unsuccessful females (3.5 ± 0.08 pups, *N* = 139; 3.3 ± 0.2 pups, *N* = 40; respectively; Tukey difference = −0.19, CI −0.72 to 0.35, *P *=* *0.81). Similarly, females that failed during gestation had nearly the same reproductive success at birth in the following year as females that bred successfully the previous year (3.3 ± 0.2 pups, *N* = 19, 3.5 ± 0.08 pups, *N* = 139, Tukey difference = 0.003, CI −0.56 to 0.56, *P *=* *0.99). Finally, time of reproductive failure in the previous year did not significantly influence a female's reproductive success at birth in the following year (failed during gestation = 3.5 ± 0.2 pups, *N* = 19, failed during lactation = 3.3 ± 0.2 pups, *N* = 21, Tukey difference = −0.19, CI −0.91 to 0.54, *P *=* *0.91).

**Table 2:** Probability of reproductively mature (≥3 years old) female Columbian ground squirrels of belonging to a given reproductive group from one year to the next

Current year	Next year			
	Breeders, %	*N*	Failed breeders, %	*N*
Breeders	78.9	172	21.1	46
Failed breeders	82.0	50	18.0	11

**Figure 2:**
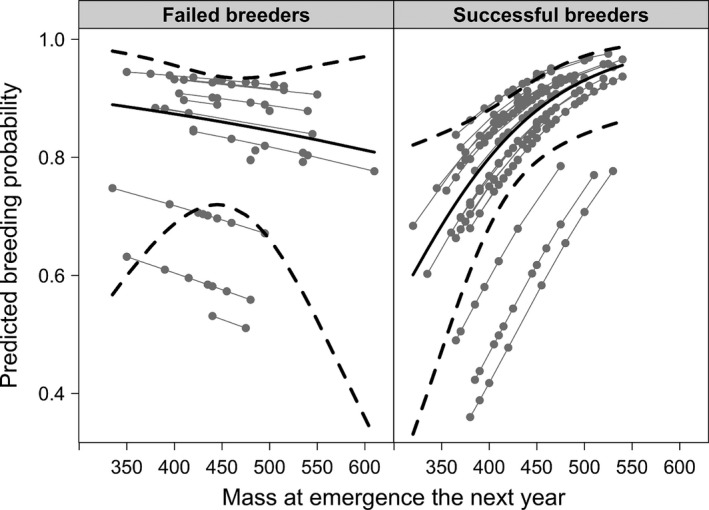
For female Columbian ground squirrels (3 years of age and older), predicted probability by year (light gray lines, each dot represents an individual) of transitioning into breeder class from previous year's breeding status (left = failed breeders, *N* = 99, right = successful breeders, *N* = 321) based on emergence mass in the next year. Solid lines are average breeding probability with 95% confidence interval dashed lines.

To examine whether success or failure in 1 year influenced the probability of successful breeding in the following year, we examined the predicted breeding probability for females of different emergence mass in the same year (Fig. 2). Changes in emergence mass the year after successfully breeding or failing, significantly influenced whether previously successful breeders maintained their breeding status or became failed breeders in the following year (mixed model, emergence mass in the following year as a fixed factor, year and female identity as random variables, binomial error distribution, $R_{\mathrm{conditional}}^{2}$ = 0.273, *N* = 218, χ^2^ = 8.19, *P* = 0.0004). For females that failed to wean offspring in the previous year, success in the following year did not depend on emergence body mass (mixed model, data subset with only failed breeders [*N* = 38], emergence mass in the following year as a fixed factor, year as a random variable, binomial family, $R_{\mathrm{conditional}}^{2}$ = 0.047, likelihood ratio test, χ^2^ = 0.33, *P* = 0.56). Litter size at birth and weaning in the year following successfully breeding or failing were significantly associated with emergence body mass in that year (mixed model, random female identity and year; $R_{\mathrm{conditional}}^{2}$ = 0.384, *N* = 180, likelihood ratio test, χ^2^ = 21.4, *P *<* *0.0001; $R_{\mathrm{conditional}}^{2}$ = 0.111, *N* = 222, likelihood ratio test, χ^2^ = 13.0, *P *<* *0.0001; respectively). Litter size at birth and weaning in the year following successfully breeding or failing were also significantly associated with changes in body mass from one spring to the next (mixed model, female identity and year as random variables; $R_{\mathrm{conditional}}^{2}$ = 0.375, *N* = 180, likelihood ratio test, χ^2^ = 21.4, *P *<* *0.0001; $R_{\mathrm{conditional}}^{2}$ = 0.117, *N* = 222, likelihood ratio test, χ^2^ = 13.0, *P *<* *0.0001; respectively). These patterns did not differ significantly between females that were successful or that failed in reproduction in the previous year, nor was there a significant interaction between breeding status and spring mass in the following year; analyses are not shown.

## Discussion

In this study, we tested for potential costs of reproduction in Columbian ground squirrels by comparing the survival and future reproduction of females that bred successfully or not in given years. Our results provide little evidence of substantial “costs” to reproduction for females in traditional fitness measures.

Although we expected future survival to be higher for females not reproducing in a given year, we found that survival was actually similar between successful and unsuccessful females. Females that failed to give birth had poorer survival (though not significantly) than successfully reproducing females. Those results confirm previous findings having failed to detect a long‐term survival cost to experimental manipulations of female reproductive effort via litter size manipulation (Hare and Murie 1992; Skibiel et al. 2013; but see contrasting results over shorter time periods, Neuhaus 2000). Our results were also similar to those of females of other Sciurid species (North American red squirrels [*Tamiasciurus hudsonicus*], Descamps et al. 2009; Fletcher et al. 2015; yellow ground squirrels [*Spermophilus fulvus*], Vasilieva and Tchabovsky 2014).

We also predicted that, when compared to unsuccessful breeders, successful females in a given year should experience poorer reproduction in the next year. However, we found little difference in the likelihood of future breeding in the following year for females that successfully reproduced or failed to wean young in the previous year, although the latter group was slightly more likely to be successful at future reproduction. There was also no clear difference in litter size during the following year for females that were previously successfully reproductive versus those that previously failed. These results confirm comparative and experimental studies that reveal little or no influence of litter size in 1 year with litter size in the next, whether the initial litter size was artificially manipulated or not (Murie and Dobson 1987; Hare and Murie 1992; Neuhaus 2000; Skibiel et al. 2013).

**Figure 3:**
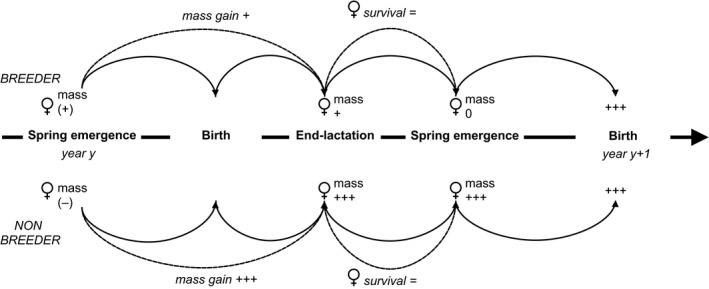
A flow diagram of mass changes for breeding and failed breeding female Columbian ground squirrels (3 years of age and older), including reproductive and survival information. From the left‐hand side of the diagram, lighter females are more likely to fail to give birth (reproduction = 0); at weaning (the end of lactation), females that have failed have gained significantly more mass than successful breeders; and both successful and unsuccessful females survive about equally well to the next spring. In the next spring, previously successful females are at about the same body mass as the previous year, but females that failed in the previous year have gained body mass; both groups of females are equally likely to be successful breeders (this probability is body mass dependent for previously successful breeders, but not for those that previously failed; this latter result not shown).

Despite no apparent negative fitness cost to breeding, allocation of resources clearly differed between females of different breeding status (see flow diagram; Fig. 3). First, females that failed during gestation were significantly lighter in body mass at spring emergence from hibernation than successful females, suggesting they lacked sufficient capital reserves to produce offspring. Stored capital at spring emergence from hibernation is known to strongly influence subsequent reproductive success in Columbian ground squirrels (Dobson et al. 1999; Broussard et al. 2003). By the time of weaning, unsuccessful females (those that failed during gestation or lactation) had gained significantly more body mass than successful females. While this difference was slightly reduced by the following spring emergence from hibernation, unsuccessful females gained close to 9% in body mass from the previous spring, while the mass of successfully reproducing females was virtually unchanged. Thus, while there appeared to be no fitness costs to breeding in these ground squirrels (see also Murie and Dobson 1987; Hare and Murie 1992; Skibiel et al. 2013), there were consequences for body mass dynamics.

For females that successfully bred, the likelihood of success in the subsequent year depended strongly on capital resources at spring emergence from hibernation, with lighter females more likely failing to repeat as successful breeders. Experimental results suggest advantages in body mass for females that forego reproduction (Hare and Murie 1992; Neuhaus 2000; Skibiel et al. 2013). This pattern, however, was not evident for females that had failed to wean litters, as their success was virtually independent of their spring body mass. Thus, the success or repeated failure of previously nonbreeding females must depend on factors other than body mass. Other factors that might influence reproductive success include daily resource income, variation in the richness of the habitat, predation, and weather patterns (e.g., Dobson and Murie 1987; Karels et al. 2000; Lane et al. 2012).

In the spring following a breeding failure, females gained significantly more body mass from the previous year than reproductively successful females. While this did not improve their subsequent likelihood of success, it resulted in these females being heavier at spring emergence, as compared to the previous year. This might explain why females that had previously failed at breeding did not subsequently show a dependency of reproductive success on body mass in the next year, as the females that reproduced successfully did (Fig. 2). All females, however, showed a dependence of litter size on body mass in the subsequent year, such that heavier females had larger litters, as also found by Risch et al. (1995) in an earlier study. As previously failed breeders gained more body mass on average from the previous year and were heavier in the next spring (Fig. 1), they were in a better position to produce larger litters. Thus, the body mass “carryover” effect after failure to reproduce may have augmented subsequent reproduction; not to a greater level than continuously reproductive females, but to a commensurate level with them. The only advantage to current failure was a chance to recoup the condition necessary to be reproductively successful in terms of producing a litter. Thus, failing females exhibited a “catch‐up” strategy, in which their capital for breeding was higher than in the previous year and commensurate with previously successful females.

In conclusion, our results fail to support the hypothesis of short‐term costs to reproduction for fully grown female ground squirrels, but demonstrate that there were consequences to successful breeding in terms of a lack of gain in body mass and perhaps body condition. Columbian ground squirrels are predominately income breeders that use “capital” (stored resources) to increase their likelihood of future reproduction (Broussard et al. 2005). Females with a heavier weaning mass proceeded to a heavier emergence mass in the next year and a higher chance of producing greater numbers of offspring. To some extent, failing to breed in 1 year allowed females to prepare for reproduction in the next year by accumulating extra energy reserves that, provided “carryover” benefits in terms of body mass, likely augmented fitness. Our study is unique in that, by comparing naturally successful and unsuccessful breeders over a 24‐year period, we highlight important “carryover” effects in terms of body mass for unsuccessful females that increased their propensity for future reproduction. Future research is needed to test the hypothesis that maternal capital and income influence resource allocations to specific offspring.

## Conflict of Interest

None declared.
